# The long non-coding RNA HOXA11-AS promotes epithelial mesenchymal transition by sponging miR-149-3p in Colorectal Cancer

**DOI:** 10.7150/jca.49809

**Published:** 2020-08-18

**Authors:** Dong Chen, Min Zhang, Jian Ruan, Xiaolin Li, Saisai Wang, Xiaofei Cheng, Huiying Zhao, Ying Zeng, Jingjing Liu, Kangxin He, Peng Zhao

**Affiliations:** 1 Department of Colorectal Surgery, First Affiliated Hospital, College of Medicine, Zhejiang University, Hangzhou 310003, Zhejiang Province, China.; 2 College of Medicine, Zhejiang University, Hangzhou 310058, Zhejiang Province, China.; 3 Department of Medical Oncology, First Affiliated Hospital, College of Medicine, Zhejiang University, Hangzhou 310003, Zhejiang Province, China.; 4 Department of Emergency, First Affiliated Hospital, College of Medicine, Zhejiang University, Hangzhou 310003, Zhejiang Province, China.; 5 State Key Laboratory for Diagnosis and Treatment of Infectious Diseases, National Clinical Research Center for Infectious Diseases, Collaborative Innovation Center for Diagnosis and Treatment of Infectious Diseases, First Affiliated Hospital, College of Medicine, Zhejiang University, Hangzhou 310003, Zhejiang Province, China.

**Keywords:** colorectal cancer (CRC), long non-coding RNA (lncRNA), HOXA11-AS, miR-149-3p, epithelial mesenchymal transition (EMT)

## Abstract

**Background:** Metastasis is the primary cause of death in colorectal cancer (CRC); the underlying mechanisms remain partly unknown. In this study, we aim to investigate the value of HOXA11-AS in survival evaluation and the potential role of HOXA11-AS/miR-149-3p axis in the CRC metastasis.

**Methods:** The expressions of HOXA11-AS, both in obtained CRC samples and adjacent noncancerous tissues, were analyzed in survival evaluation. Competing endogenous RNAs (CeRNAs) Analysis were employed to reveal the potential relationship between HOXA11-AS and miR-149-3p. It was further confirmed by Quantitative real-time polymerase chain reaction (qRT-PCR) and Dual-luciferase reporter assay. Migration and invasion assay were used to verify the potential role of HOXA11-AS and miR-149-3p in the regulation of CRC metastasis. The potential pathway was explored by Western blot analysis.

**Results:** The expression of HOXA11-AS in the CRC tissue is significantly higher than the expression in adjacent noncancerous tissue (p<0.0001). High expressions of HOXA11-AS were noticeably correlated with clinicopathologic characteristics including advanced clinical stage (p=0.021), larger tumor size (p<0.001) and frequent tumor recurrence (p=0.001). The overall survival in HOXA11-AS-High group was significantly shorter than the HOXA11-AS-Low group (p<0.001). Advanced clinical stage, tumor size and high expression of HOXA11-AS were showed as independent prognostic prediction factors for the 5-year tumor relapse of CRC patients (p<0.001). HOXA11-AS acts as a potential molecular sponge for miR-149-3p, in the promotion of CRC metastasis. In the miR-149-3p mimic-treated group, the expression of E-cadherin was increased, whereas the expression of N-cadherin, Snail, Slug, TGF-β1, Wnt2b, Twist and C/EBPβ was decreased.

**Conclusion:** This study demonstrates that high expression of HOXA11-AS is correlated with CRC progression and poor prognosis and may promote metastasis via EMT by modulating miR-149-3p.

## Introduction

Colorectal cancer (CRC) is the third most common cancer in the United States, according to 2019 statistics for both male and female cancers 1, and it was the second most common cancer in China in 2018 2. Although great progress has been made in the past several decades related to the prevention, detection, and treatment of CRC, metastasis is still the primary cause of death 3, and the underlying mechanisms remain partly unknown 4.

Accumulating evidence has demonstrated that long non-coding RNAs (lncRNAs) and microRNAs (miRNAs) play important roles in tumor growth and metastasis and are linked to the activation of oncogenic signaling pathways or inactivation of tumor-suppressive signaling in the nucleus 4, 5. Although most functions of lncRNAs and miRNAs have not been clarified, recent evidence indicates that they are involved in the regulation of CRC proliferation and metastasis 6.

The expression of homeobox A11 antisense RNA (HOXA11-AS), a lncRNA, has been found in diverse human neoplasms, such as glioma, epithelial ovarian cancer, lung adenocarcinoma, gastric cancer, hepatocellular carcinoma, uterine cervix carcinoma, breast cancer, and CRC 7-15. In our previous study, HOXA11-AS was found to function as a competing endogenous RNA (ceRNA) to regulate peptidyl arginine deiminase 2 expression by sponging miR-125a-5p, promoting the metastasis of CRC 15. It was reported that some lncRNAs may be related to several miRNAs in ceRNA regulation of CRC 16. In our previous study, many associated miRNAs including miR-149-3p were found to potentially participate in the function of HOXA11-AS as a ceRNA regulator of CRC metastasis.

In this study, we evaluated whether HOXA11-AS is associated with the prognosis of CRC, as well as the effect and mechanism of the HOXA11-AS/miR-149-3p axis in the regulation of CRC metastasis.

## Materials and Methods

### Human tissue samples and cell lines

Tissue samples were collected from patients visiting the Department of Colorectal Surgery, First Affiliated Hospital, Zhejiang University (Hangzhou, China). The first 105 CRC samples were collected between June 2014 and September 2014; the second 30 primary CRC samples were obtained from 15 patients with CRC and liver metastasis and 15 patients with CRC without metastasis between April 2016 and September 2016. None of the patients received neoadjuvant therapy. Surgical staging was based on the tumor/node/metastasis (TNM) system. The inclusion criteria include (1) histologically confirmed diagnosis and (2) no previous treatment. The exclusion criteria include (1) serious complications, (2) presence of other malignant diseases, or (3) incomplete follow-up data. For staging, Cancer staging system defined by the United States Joint Commission 8th edition was adopted. The CRC samples were postoperative pathologically confirmed as colorectal adenocarcinoma, and the liver samples were confirmed to be metastatic adenocarcinoma by surgery or biopsy. Samples were collected within 10 min of tumor excision, immersed in RNA later™ Stabilization Solution (Thermo Fisher Scientific, Waltham, MA, USA) immediately, and stored at -80°C until use. Written informed consent was obtained from all patients. This study was approved by the Ethics Committee of First Affiliated Hospital, College of Medicine, Zhejiang University. The human CRC cell lines SW480, SW620, HCT116, HT-29, and RKO were obtained from Tongpai Biological Technology (Shanghai, China). SW480, SW620, HT-29, and HCT116 cells were cultivated in RPMI-1640 medium (Gibco, Carlsbad, CA, USA) supplemented with 10% fetal bovine serum (FBS; HyClone, Logan, UT, USA). RKO cells were cultivated in Minimal Essential Media (Gibco) supplemented with 10% FBS. All cells were maintained in a 37°C incubator with a humidified atmosphere containing 5% CO_2_.

### RNA extraction and qRT-PCR

Total RNA was isolated using TRIzol reagent (Invitrogen, Carlsbad, CA, USA) according to the manufacturer's instructions. RNA samples were reverse transcribed into cDNA using different primers and the Prime Script Kit (Takara Bio Inc., Otsu, Japan). qRT-PCR was performed in triplicate to amplify lncRNAs and miRNAs using the SYBR Premix Ex Taq™ Kit (Takara Bio Inc.). The expression levels were normalized to that of β-actin, and relative gene expression was calculated as ΔCt = Ct_gene_ - Ct_reference_. The fold change in gene expression was calculated using the 2^-ΔΔCt^ formula. The primer sequences are shown in **Table 1.**

### Dual-luciferase reporter assay

Luciferase constructs harboring wild-type (WT) HOXA11-AS or mutant (Mut) HOXA11-AS, which contained a mutation site that abolishes targeting by miR-149-3p, were generated to analyze the interaction between HOXA11-AS and miR-149-3p. 293T cells were seeded onto 24-well plates and co-transfected with a luciferase reporter and miR-149-3p or the negative control (NC). Cells were lysed according to the instructions provided with the Dual-Luciferase Reporter Assay System (Promega, Madison, WI, USA). Luciferase activity was measured 48 h after transfection using the Panomics Luminometer (Affymetrix, Santa Clara, CA, USA), according to the manufacturer's instructions. Luciferase activity was normalized to that of Renilla luciferase.

### Construct generation and transient transfection

A small interfering RNA (siRNA) against HOXA11-AS (HOXA11-AS-siRNA), NC siRNA, miR-149-3p inhibitor, and miR-149-3p mimic were obtained from Gene Pharma (Shanghai, China). The HCT116 human CRC cells (2 × 10^5^ cells) were transfected with these constructs at a final concentration of 25 nmol/L using Lipofectamine 2000 Reagent (Life Technologies, Carlsbad, CA, USA). Cells were transfected with the pcDNA-HOXA11-AS constructs at a final concentration of 1 μg/μL according to the manufacturer's protocol. After transfection for 48 h, total RNA was isolated from the harvested cells using TRIzol reagent (Invitrogen). Empty pEX3 vector and scrambled sequences of the miR-149-3p mimics, miR-149-3p inhibitor, or siRNAs were used as NCs.

### Scratch assay

HCT116 cells were seeded at 4.0 × 10^5^/mL. After 48 h, confluent cells were linearly scratched using a 20 μL pipette chip. The scratched region was photographed immediately, 24 h and 48 h after scratching by a microscope equipped with a camera. The photograph was traced to tracing paper, followed by the coloring of cells using image editing software (FireAlpaca, Tokyo, Japan). Subsequently, the area containing cells as a percentage of the total area was determined using the area measurement function of VHX-5000 (Keyence, Osaka, Japan). LIPUS exposure at a frequency of 3 MHz and intensities of 160 and 240 mW/cm^2^ was performed immediately, 24 h, and 48 h after scratching.

### Migration and invasion

Cells were trypsinized and seeded at 50,000/well on both Matrigel-coated (Corning, Tewksbury, MA, USA) and uncoated (BD Biosciences, San Jose, CA, USA) Transwell filters in a 24-well plate. Both groups of cells were allowed to migrate or invade for 12 h toward the lower chambers containing 10% FBS. Three-Step-Stain (Richard-Allan Scientific, San Diego, CA, USA) was applied when the cells migrated or invaded through the uncoated or coated filters. Each filter was counted entirely under the rule of four 10× fields, whereas migration or invasion was quantified as the fold change relative to the control.

### Western blot analysis

Cell lysates were homogenized in Cell Lysis Solution (Sigma-Aldrich, St. Louis, MO, USA) and centrifuged at 4ºC for 5 min. Samples were subjected to sodium dodecyl sulfate-polyacrylamide gel electrophoresis for 3 h and then electrotransferred to polyvinylidene fluoride membranes (Amersham Biosciences, Piscataway, NJ, USA) for an additional 2 h. Antibodies against transforming growth factor beta 1 (TGF-β1), CCAAT-enhancer binding protein beta (C/EBPβ), Snail 1, Wnt2b, Slug, Twist, E-cadherin, and N-cadherin were used for Western blot analysis. The Snail 1 antibody was purchased from Biorbyt Ltd. (Cambridge, UK); the other antibodies were purchased from Abcam (Cambridge, MA, USA). After incubation with a specific antibody against GAPDH at 4ºC overnight, the membranes were washed with 1% Tris-buffered saline containing Tween 20 in triplicate, incubated with the appropriate secondary antibodies for 1 h, and detected by chemiluminescence.

### Statistical analysis

All statistical analyses were performed by SPSS 16.0 software (SPSS Inc., Chicago, IL, USA). Significant differences between two groups were estimated by Student's *t*-test, Pearson's chi-square test or Wilcoxon signed-rank test as appropriate. Pearson's correlation analysis was used to estimate the relationship between the expression of HOXA11-AS and that of miR-149-3p. *p*<0.05 was considered statistically significant.

## Results

### HOXA11-AS expression is correlated with CRC progression and poor prognosis

**Figure 1:**
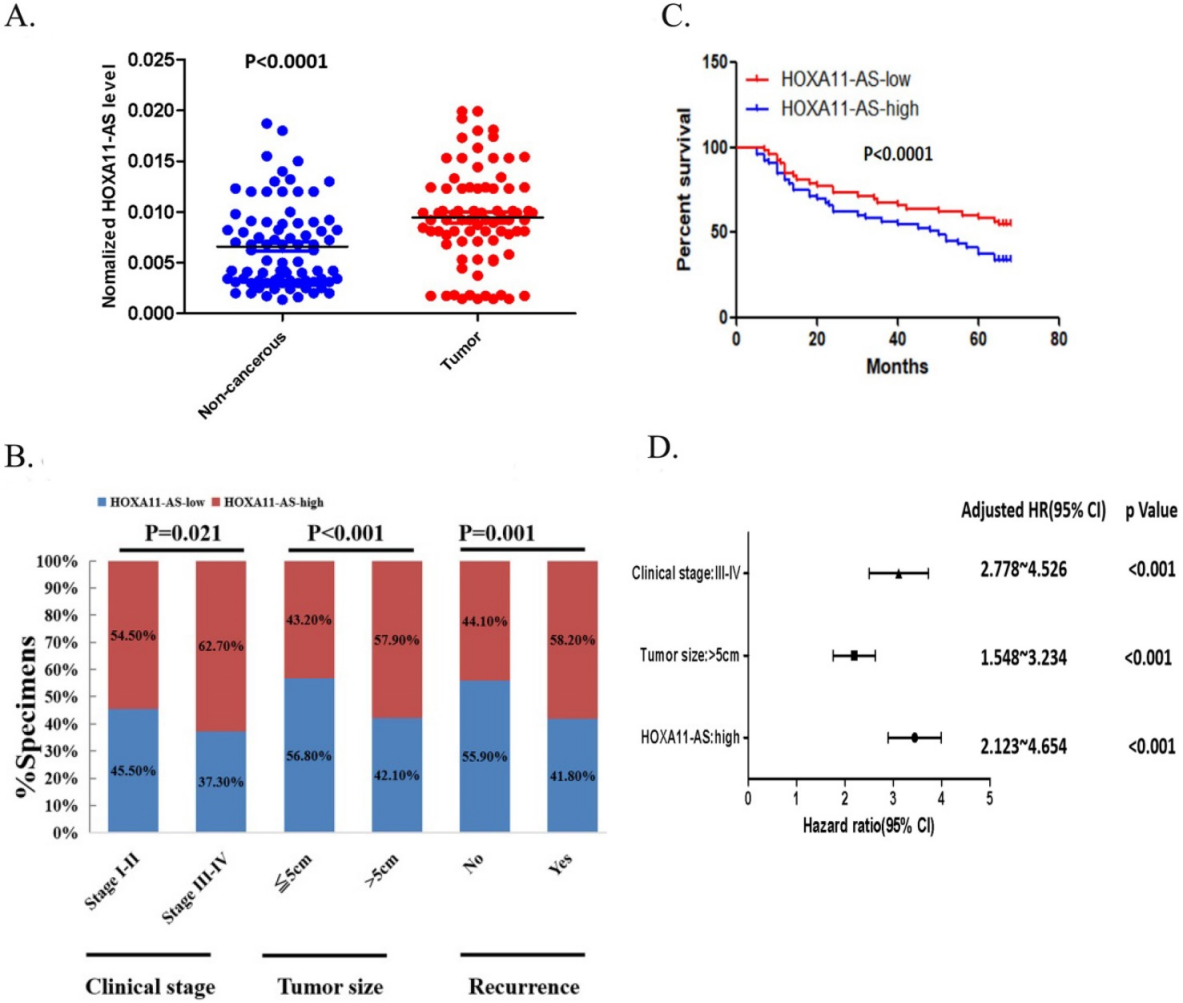
** HOXA11-AS expression is correlated with colorectal cancer (CRC) poor prognosis.** (**A**) The expression of HOXA11-AS in the CRC tissue is significantly higher than the expression in adjacent noncancerous tissue (*p<*0.0001). (**B**) High expressions of HOXA11-AS were noticeably correlated with clinicopathologic characteristics including advanced clinical stage (*p=*0.021), larger tumor size (*p<*0.001) and frequent tumor recurrence (*p=*0.001). (**C**) The overall survival in HOXA11-AS-High group was significantly shorter than the HOXA11-AS-Low group (*p<*0.001) in the Kaplan-Meier survival curves. (**D**) Advanced clinical stage, tumor size and high expression of HOXA11-AS were showed as independent prognostic prediction factors for the 5-year tumor relapse of CRC patients (*p<*0.001).

We investigated the clinical significance of HOXA11-AS expression in CRC tissue samples by qRT-PCR. The 105 patients were classified into high and low HOXA11-AS expression groups, according to the median level of relative HOXA11-AS expression in the tumor tissues. As shown in **Figure 1A**, the expression of HOXA11-AS was significantly upregulated in CRC tissues compared with adjacent noncancerous tissues. Furthermore, high expression of HOXA11-AS was noticeably correlated with clinicopathologic characteristics including advanced clinical stage, large tumor size, and frequent tumor recurrence (**Fig. 1B**). To evaluate the relationship between HOXA11-AS expression and CRC prognosis, a Kaplan-Meier survival curve was generated. Patients in the high HOXA11-AS group had significantly shorter overall survival compared with those in the low HOXA11-AS group (*p*<0.001; **Fig. 1C**). Moreover, advanced clinical stage, large tumor size, and high expression of HOXA11-AS were independent prognostic factors for the 5-year relapse-free survival of CRC patients (**Fig. 1D**).

### The potential role of the HOXA11-AS/miR-149-3p axis in CRC metastasis

**Figure 2:**
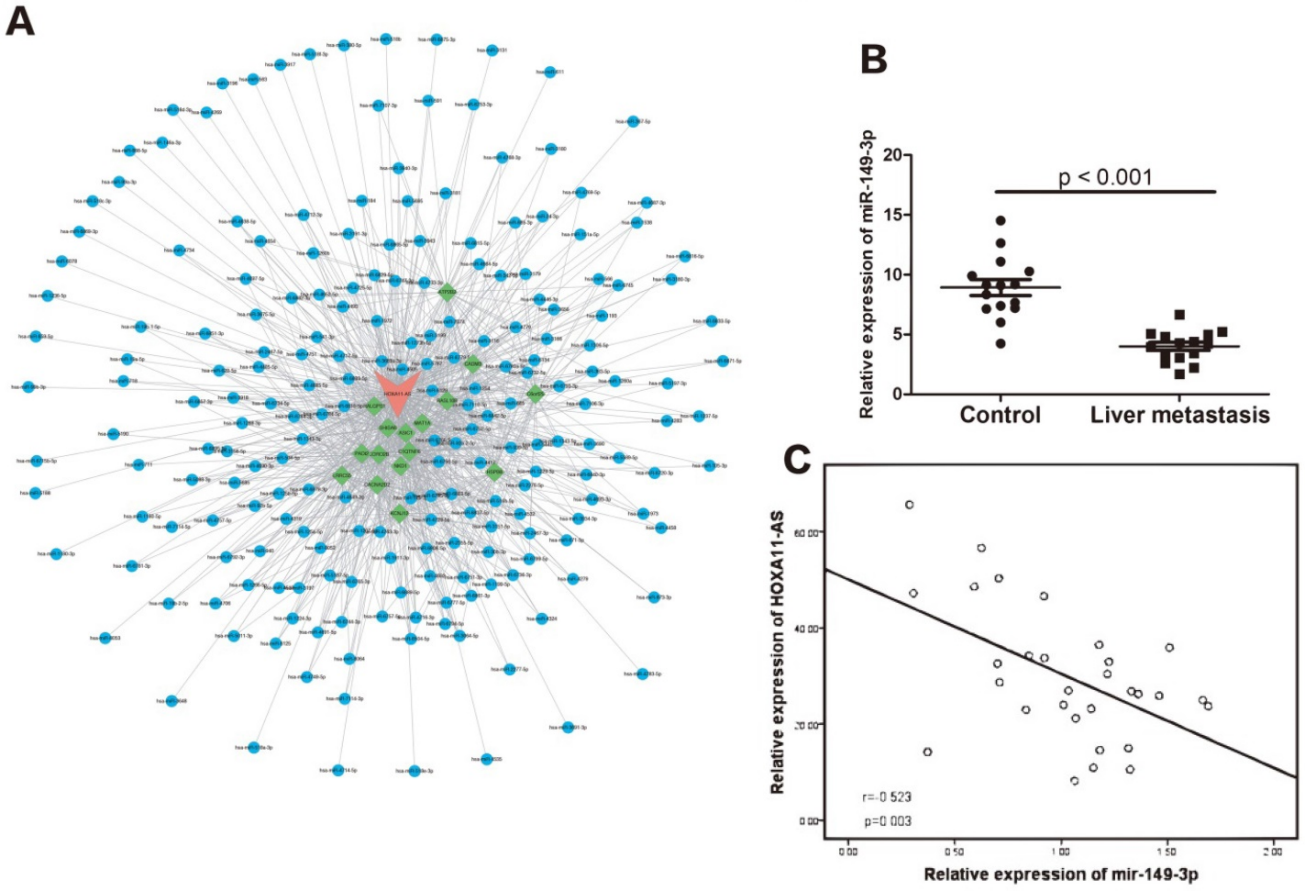
** The potential function of HOXA11-AS/miR-149-3p axis in the regulation of colorectal cancer (CRC) liver metastasis.** (**A**) The competing endogenous RNAs (CeRNAs) analysis showed the potential **HOXA11-AS/miR-149-3p pathway.** (**B**) The expression of miR-149-3p in the primary CRC tissue (with liver metastasis) was significantly lower than in the CRC tissue without metastasis (*p<*0.001). (**C**) The expression of miR-149-3p was negative correlated with the expression of miR-149-3p in CRC tissues (*p=*0.003).

We performed bioinformatics analysis using Starbase 2.0 (http://starbase.sysu.edu.cn) and found that the HOXA11-AS/miR-149-3p axis may function in the regulation of CRC metastasis (**Fig. 2A**). Compared with the 15 samples of CRC without metastasis, the relative expression of miR-149-3p was significantly lower in the 15 CRC samples with liver metastasis (*p<*0.001), as shown in **Figure 2B.** Moreover, a negative correlation was found between the relative expression of miR-149-3p and that of HOXA11-AS (**Fig. 2C**).

### HOXA11-AS acts as a molecular sponge for miR-149-3p

**Figure 3:**
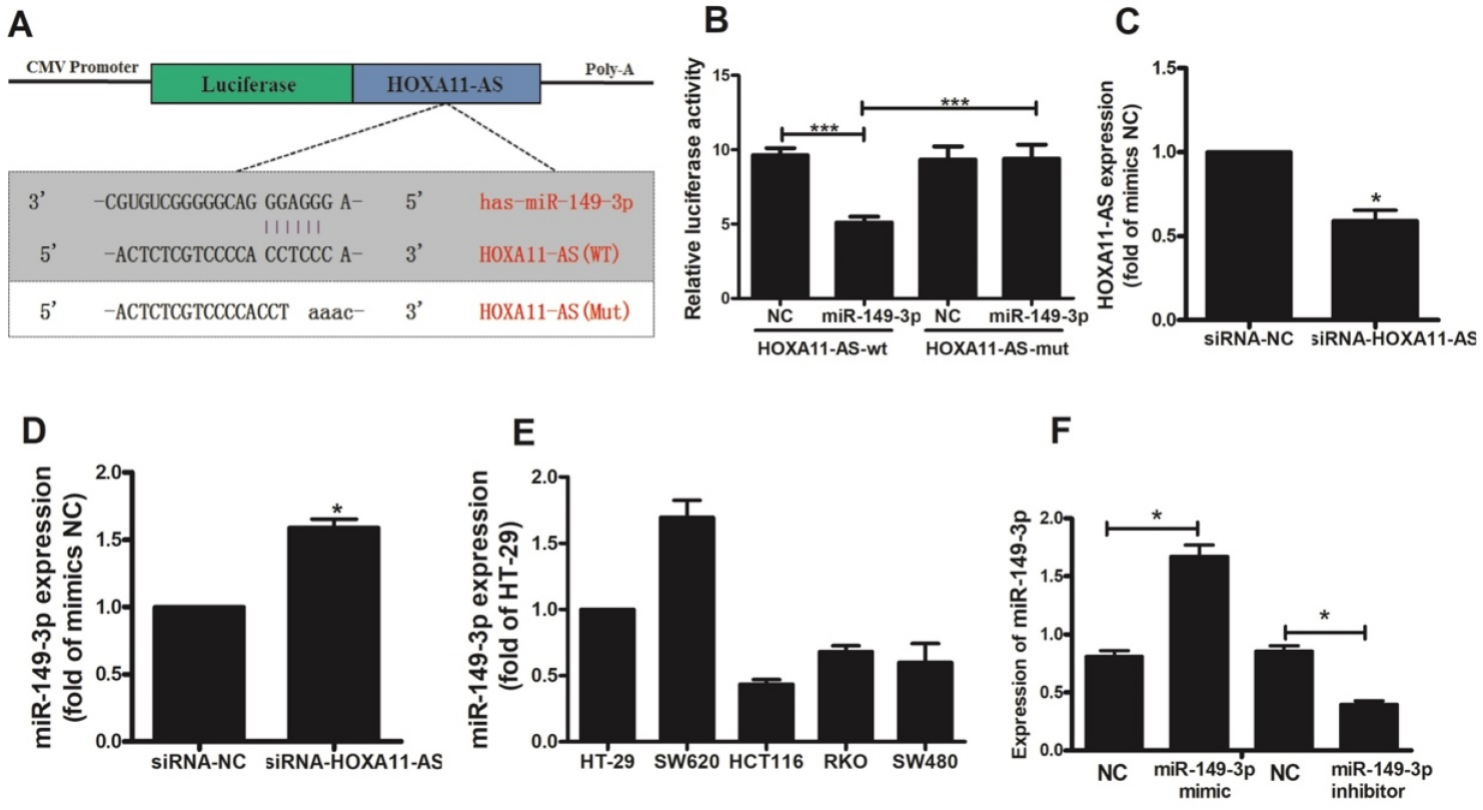
** HOXA11-AS acts as a molecular sponge for miR-149-3p.** (**A**) The putative miR-149-3p-binding sequence of HOXA11-AS. A mutation was generated in the HOXA11-AS sequence in the complementary site for the seed region of miR-149-3p. (**B**) Verification of the target relationship between miR-149-3p and HOXA11-AS. (**C**) HOXA11-AS siRNA transfection into HCT116 cells significantly reduced the expression of HOXA11-AS (*p=*0.0226). (**D**) HOXA11-AS siRNA transfection into HCT116 cells significantly increased the expression of miR-149-3p (*p=*0.011). (**E**) The relative expression of miR-149-3p in colorectal cell lines HT-29, SW620, HCT116, RKO and SW480. (**F**) Verification of the effectiveness of miR-149-3p mimic and miR-149-3p inhibitor.

Based on our ceRNA analysis, we found that HOXA11-AS and miR-149-3p contained complementary base pairs (**Fig. 3A**), indicating that HOXA11-AS may act as a sponge to deregulate miR-149-3p. Thereafter, we generated luciferase reporter constructs harboring WT or Mut HOXA11-AS as the binding sites for investigation. Luciferase assays indicated a significant reduction in luciferase activity after co-transfection of miR-149-3p and a WT HOXA11-AS vector, but not Mut HOXA11-AS (*p<*0.0001; **Fig. 3B**). The transfection of HOXA11-AS-siRNA in HCT116 cells was validated by qRT-PCR. HOXA11-AS-siRNA caused a significant decrease (*p*=0.0226) in HOXA11-AS expression (**Fig. 3C**). The effect of HOXA11-AS on the endogenous expression of miR-149-3p was further examined. qRT-PCR revealed that knockdown of HOXA11-AS substantially increased the expression of miR-149-3p in HCT116 cells (*p*=0.011; **Fig. 3D**). HOXA11-AS expression was detected in all five CRC cell lines (**Fig. 3E**), and the HCT116 cell line was selected for subsequent analysis because its expression level was lowest. We also constructed a miR-149-3p mimic and miR-149-3p inhibitor and evaluated their effects by qRT-PCR (**Fig. 3F**).

### The HOXA11-AS/miR-149-3p axis regulates the metastatic potential of CRC cells

**Figure 4:**
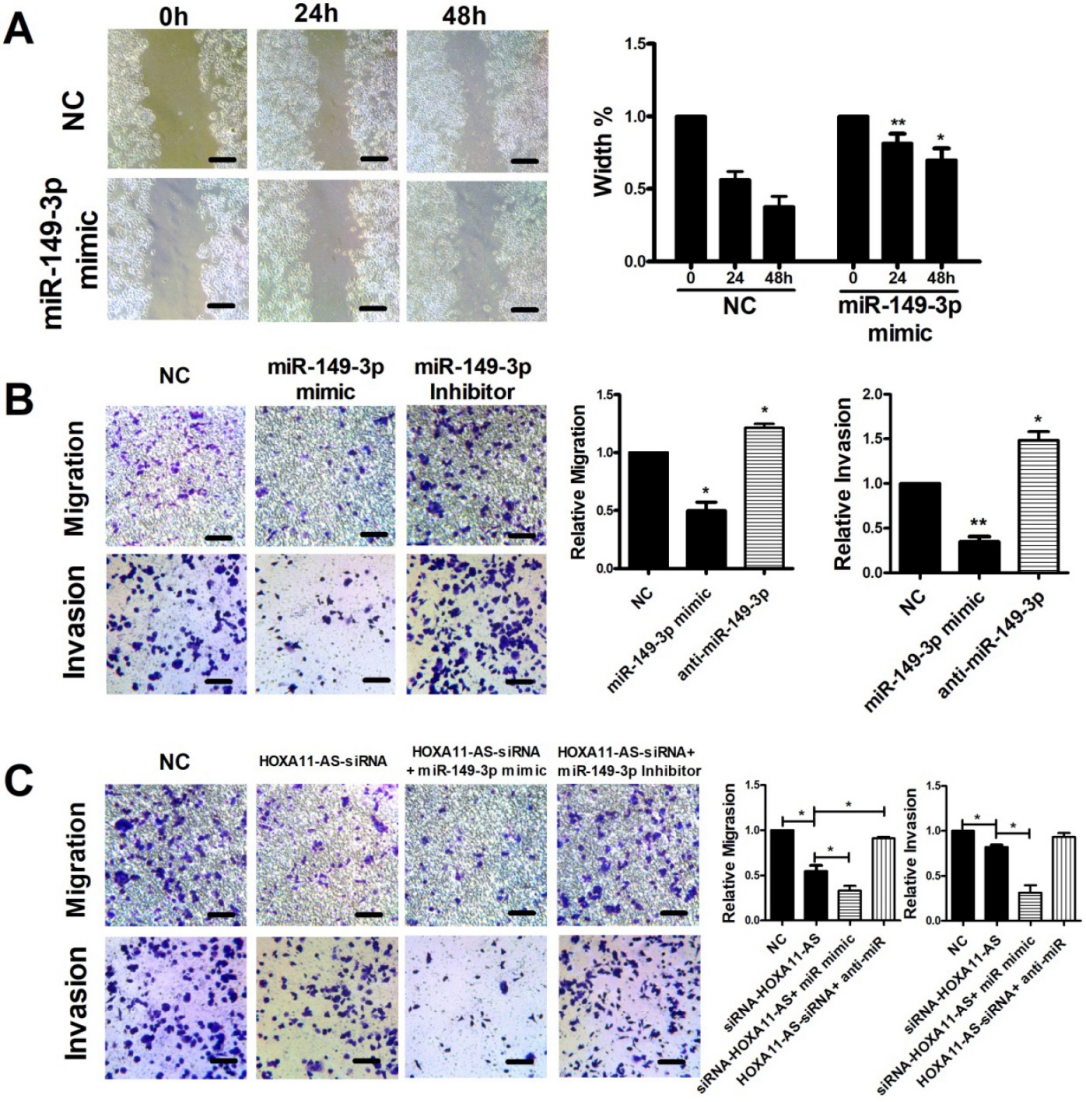
** HOXA11-AS/miR-149-3p axis regulated the metastasis of colon cancer cell.** (**A**) Representative images of the scratch area at 0, 24 and 48 hours post-treatment are shown. Wound closure became significantly lower both 24 and 48h after miR-149-3p treated. (**B**) miR-149-3p mimic inhibited the migration and invasion of HCT116 cells significantly; while miR-149-3p inhibitor increased the migration and invasion of HCT116 cells significantly. (**C**) The significant effectiveness of HOXA11-AS/miR-149-3p axis in regulating migration and invasion of HCT116 cells. *: *p<*0.05, **: *p<*0.01. Scale bar: 50 µm.

Scratch, migration, and invasion assays were performed to verify the functional roles of miR-149-3p and the HOXA11-AS/miR-149-3p axis in the regulation of CRC metastasis. The scratch assay indicated that overexpression of miR-149-3p markedly reduced the migration of HCT116 cells (**Fig. 4A**). Moreover, a Transwell assay demonstrated that overexpression of miR-149-3p significantly inhibited cell migration and invasion, whereas miR-149-3p inhibitors promoted migration and invasion of HCT116 cells (**Fig. 4B**). To further investigate the role of the HOXA11-AS/miR-149-3p axis in the regulation of CRC metastasis, we transfected the NC siRNA, HOXA11-AS-siRNA, HOXA11-AS-siRNA plus the miR-149a-3p mimic, and HOXA11-AS-siRNA plus the miR-149a-3p inhibitor into HCT116 cells. Both the migration and invasion of HCT116 cells were affected by HOXA11-AS-siRNA and further affected by the miR-149a-3p mimic or miR-149a-3p inhibitor (**Fig. 4C**).

### Effects of miR-149-3p on the expression of TGF-β1, C/EBPβ, Snail 1, Wnt2b, Slug, Twist, E-cadherin, and N-cadherin *in vitro*

**Figure 5:**
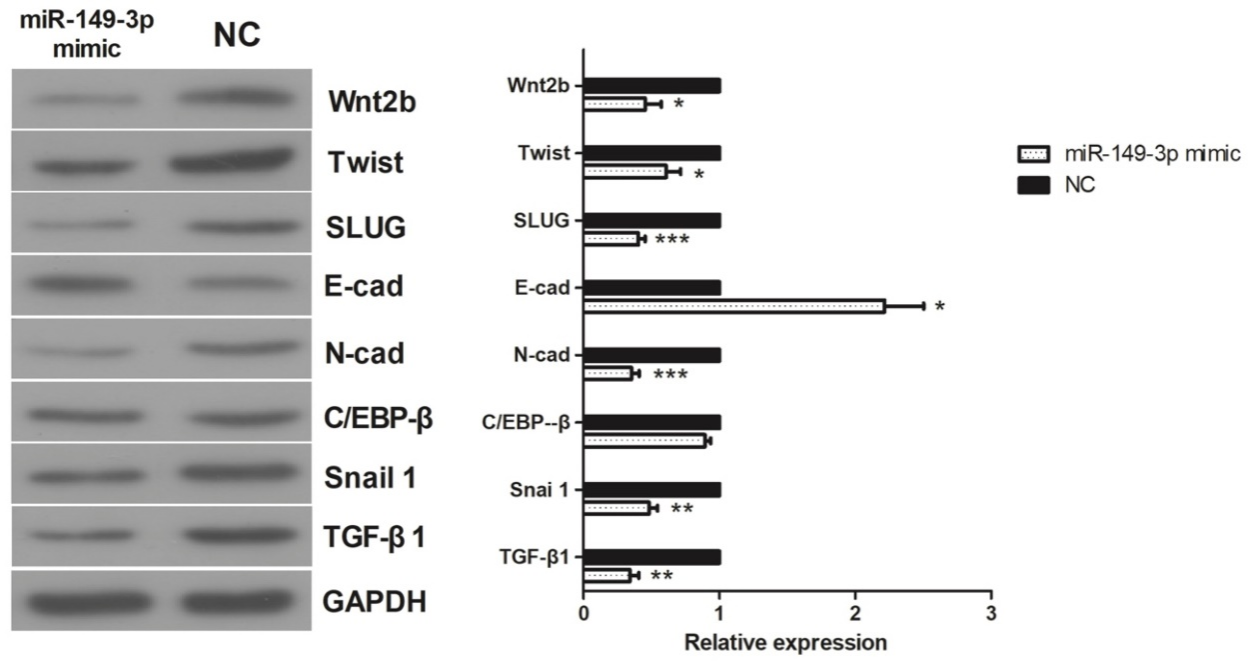
** Western Blot results of TGF-β1, C/EBP β, Snail 1, Wnt 2b, Slug, Twist, E-cadherin and N-cadherin.** In miR-149-3p mimics treated group, the expression of E-cadherin was significantly increased; the expression of N-cadherin, Snail, Slug, TGF-β1, Wnt2b and Twist were significantly decreased; the expression of C/EBPβ had no significant difference. *: *p<*0.05, **: *p<*0.01, ***: *p<*0.001.

The activities of TGF-β1, C/EBPβ, Snail 1, Wnt2b, Slug, Twist, E-cadherin, and N-cadherin were assessed in HCT116 CRC cells. As shown in **Figure 5**, in the miR-149-3p mimic-treated group, the expression of E-cadherin was increased, whereas the expression of N-cadherin, Snail, Slug, TGF-β1, Wnt2b, Twist and C/EBPβ was decreased. These results suggest that the epithelial-mesenchymal transition (EMT) may play a crucial role in the regulation of miR-149-3p in the metastasis of CRC.

## Discussion

Although extensive studies have elucidated the genetic and epigenetic regulation of CRC oncogenesis, there is still an urgent need to improve the diagnostic and treatment approaches for CRC because of its tremendous heterogeneity and high mortality rates. To this end, establishing effective markers and targets to better understand the underlying mechanisms is of great importance.

In gastric cancer, oral squamous cell carcinoma, osteosarcoma, renal cancer, hepatocellular carcinoma, breast cancer, prostate cancer and thyroid cancer, high expressions of HOXA11-AS have been found; furthermore, the upregulated expression of HOXA11-AS were associated with tumor progression and metastasis 12-14, 17-26. Here, we observed overexpression of HOXA11-AS in CRC tissues, and high expression of HOXA11-AS was positively correlated with more aggressive clinicopathological parameters for the first time. Our results suggested that high HOXA11-AS expression in tumor tissue is an independent risk factor for HCC patients after radical resection. Moreover, a higher protein level of HOXA11-AS was correlated with poorer overall survival in CRC patients. Additionally, univariate analyses showed that higher HOXA11-AS expression, a larger tumor size, more frequent metastasis/recurrence, and an advanced TNM stage were correlated with an increased risk of death in CRC patients. Thus, HOXA11-AS appears to be a potent prognostic marker of survival in CRC.

miR-149-3p potentially plays a key role in the proliferation and metastasis of diverse neoplasms. This miRNA is reportedly upregulated in the plasma of melanoma patients compared with healthy controls 23 and is highly expressed in non-small cell lung cancer tissue 24. Meanwhile, it is downregulated in gastric cancer cells and tissue 20. miR-149-3p was found to enhance prostate cancer cell motility and invasiveness by efficiently downregulating disabled homolog 2-interacting protein, facilitating activation of nuclear factor kappa B signaling, and promoting expression of pro-inflammatory and pro-angiogenic factors 27. However, miR-149-3p was found to be a tumor inhibitor in most previous studies. For example, it reportedly inhibits pancreatic cancer by regulating the Akt1 signaling pathway 28, inhibits gastric cancer via Wnt-1 signaling or the ZEB1-AS1/miR-149-3p axis 29, 30, inhibits the proliferation, migration, and invasion of bladder cancer by targeting S100 calcium-binding protein A4 31, and inhibits renal cell carcinoma by targeting forkhead box protein M1 32. To date, there is no report about the role of miR-149-3p in CRC. In this investigation, we found that the expression of miR-149-3p was significantly decreased (*p*<0.0001) in the tissue of CRC patients with liver metastasis. Furthermore, the expression of miR-149-3p was negatively correlated with that of HOXA11-AS. Above results inferred that miR-149-3p may inhibits the metastasis of CRC via binding to HOXA11-AS.

In this investigation, we verified that miR-149-3p directly binding to HOXA11-AS in dual-luciferase reporter assay. Moreover, the negative correlation between miR-149-3p and HOXA11-AS was vevified *in vitro*. The inhibition of miR-149-3p to CRC metastasis was verified in scratch, migration and invasion assays.

EMT is a crucial process in tumor progression that affects the key steps of morphogenesis by converting epithelial cells into cells with mesenchymal attributes 33. EMT is primarily known as a phenotypic transformation process during embryonic development, wound healing, and tissue remodeling 34. Diverse factors, such as Snail*,* E-cadherin, cyclin D, TGF-β, nodal, N-cadherin, cadherins 6, 7, and 11, epidermal growth factor, TGF-βR, Notch, ErbB, and hepatocyte growth factor/scatter factor, are involved in regulating EMT via the integrin-linked kinase, Wnt, phosphoinositide 3-kinase, and Src signaling pathways 35-44. Moreover, various proteins, such as p38-interacting protein, p38 mitogen-activated protein kinase, and 4.1R, ezrin, radixin, moesin (FERM) protein.

FERM protein, metalloproteases, and extracellular signal-related kinases are involved in the process of EMT 40, 45-47. The significance of EMT in human cancers was revealed by studies that provided morphological and imaging evidence 48, 49. Accumulating evidence has shown that EMT plays a key role in tumor invasion and metastasis 34, 50. The hallmarks of EMT include decreased expression of E-cadherin, as well as increased expression of N-cadherin, Snail, Slug, Twist, TGF-β1, Wnt2b, and C/EBPβ 50-52. In this investigation, overexpression of miR-149-3p led to an increase in E-cadherin expression and decreases in N-cadherin, Snail, Slug, Twist, TGF-β1, Wnt2b, and C/EBPβ expression. Thus, miR-149-3p may inhibit EMT by targeting the abovementioned signaling pathways.

This study demonstrates that high expression of HOXA11-AS is correlated with CRC progression and poor prognosis and may promote metastasis via EMT by modulating miR-149-3p.

## Figures and Tables

**Table 1:** The sequence of the primers

Gene	Forward primer	Reverse primer
HOXA11-AS	GAGTGTTGGCCTGTCCTCAA	TTGTGCCCAGTTGCCTGTAT
miR-149-3p	CGAAAGCACGUAAUCGCCGGUGUAA	
miR-149-3p inhibitor	GCUUUCGUGCAUUAGCGGCCACAUU	
β-actin	TGAGGATGTCACGGTTCCAG	GTCACCTTCACCGTTCCAGT

## Abbreviations

CRC: colorectal cancer
lncRNA: long non-coding RNA
miRNAs: micro RNAs
CeRNAs: competing endogenous RNAs
HOXA11-AS: HOMEOBOX A11 antisense RNA
EMT: epithelial mesenchymal transition
qRT-PCR: Quantitative real-time polymerase chain reaction
